# Hypoxia-inducible factor 1 mediates TAZ expression and nuclear localization to induce the breast cancer stem cell phenotype

**DOI:** 10.18632/oncotarget.2997

**Published:** 2014-12-18

**Authors:** Lisha Xiang, Daniele M. Gilkes, Hongxia Hu, Naoharu Takano, Weibo Luo, Haiquan Lu, John W. Bullen, Debangshu Samanta, Houjie Liang, Gregg L. Semenza

**Affiliations:** ^1^ Department of Oncology and Southwest Cancer Center, Southwest Hospital, Third Military Medical University, Chongqing, China; ^2^ Vascular Program, Institute for Cell Engineering, Johns Hopkins University School of Medicine, Baltimore, MD; ^3^ McKusick-Nathans Institute of Genetic Medicine, Johns Hopkins University School of Medicine, Baltimore, MD; ^4^ Departments of Pediatrics, Medicine, Oncology, Radiation Oncology, and Biological Chemistry, Johns Hopkins University School of Medicine, Baltimore, MD; ^5^ Department of Biochemistry, School of Medicine, Keio University, Tokyo, Japan

**Keywords:** Aldefluor assay, basal-like breast cancer, mammospheres, targeted therapy, triple-negative breast cancer

## Abstract

Intratumoral hypoxia, which is associated with breast cancer metastasis and patient mortality, increases the percentage of breast cancer stem cells (BCSCs) but the underlying molecular mechanisms have not been delineated. Here we report that hypoxia-inducible factor 1 (HIF-1) triggers the expression and activity of TAZ, a transcriptional co-activator that is required for BCSC maintenance, through two discrete mechanisms. First, HIF-1 binds directly to the *WWTR1* gene and activates transcription of TAZ mRNA. Second, HIF-1 activates transcription of the *SIAH1* gene, which encodes a ubiquitin protein ligase that is required for the hypoxia-induced ubiquitination and proteasome-dependent degradation of LATS2, a kinase that inhibits the nuclear localization of TAZ. Inhibition of HIF-1α, TAZ, or SIAH1 expression by short hairpin RNA blocked the enrichment of BCSCs in response to hypoxia. Human breast cancer database analysis revealed that increased expression (greater than the median) of both TAZ and HIF-1 target genes, but neither one alone, is associated with significantly increased patient mortality. Taken together, these results establish a molecular mechanism for induction of the BCSC phenotype in response to hypoxia.

## INTRODUCTION

Breast cancer mortality occurs in patients whose cancer cells metastasize to distant sites, such as the lungs, bones, and brain. Individual cells must reach the site of metastasis and proliferate to form a secondary tumor. Only a small percentage of the cancer cells in a primary breast tumor have self-renewal capacity, which is necessary to form a metastasis or recurrent tumor, and are designated as tumor initiating cells or cancer stem cells (CSCs). Several different assays identify subpopulations of cells that are enriched for breast CSCs (BCSCs). The Aldefluor assay is based on the activity in BCSCs of the ALDH1 family of aldehyde dehydrogenases, which convert a non-fluorescent substrate into a fluorescent product that can be identified by flow cytometry [1-3]. The mammosphere assay is based on the ability of BCSCs to propagate as multicellular spheroids in suspension culture [4, 5]. Flow cytometric identification of CD44^high^/CD24^low^ cells is a useful measure of BCSCs in luminal-type breast cancer but not in basal-like breast cancer cell lines, which express *CD44* at high levels [6]. Both ALDH^+^ and mammosphere-forming cells are highly enriched for tumor-initiating BCSCs [1-6].

Several transcription factors have been implicated in the BCSC phenotype. TAZ (transcriptional co-activator with PDZ binding motif) is an effector of the Hippo pathway [7] that interacts with DNA binding proteins of the TEAD (TEA/ATTS domain) family to activate transcription of target genes, including *CTGF*, *SERPINE1*, and *BIRC5*, which encode connective tissue growth factor, plasminogen activator inhibitor 1 (PAI-1), and survivin, respectively [8-11]. TAZ is expressed in 80% of high-grade breast cancers and promotes BCSC self-renewal and tumor initiation capacity [10]. Amplification of the *WWTR1* gene, which encodes TAZ mRNA, was identified in less than 10% of breast cancers, suggesting that other mechanisms must account for increased TAZ mRNA expression in the majority of cases. TAZ is also regulated post-translationally, as phosphorylation of TAZ by the kinase LATS1 or LATS2 blocks its nuclear localization and transcriptional activity [7] and it is not clear whether or how inhibition by LATS1/2 is down-regulated in breast cancer.

Hypoxia has been shown to induce the CSC phenotype in glioma [12] and breast cancer [3, 13] through the activity of hypoxia-inducible factors (HIFs). HIF transcriptional activity is constitutively increased in mouse lymphoma and human acute myeloid leukemia CSCs, which were eliminated by treatment with a HIF-1 inhibitor [14]. HIFs are also required for the maintenance of hematopoietic stem cells [15] and for the reprogramming of differentiated human cells to induced pluripotent stem cells [16]. However, the molecular mechanisms by which HIFs contribute to the stem cell phenotype have not been determined.

HIFs are heterodimers composed of an O_2_-regulated HIF-1α or HIF-2α subunit and a constitutively expressed HIF-1ß subunit [17]. HIF-1α and HIF-2α are subject to prolyl hydroxylation, ubiquitination, and proteasomal degradation under normoxic conditions, whereas hydroxylation is inhibited under hypoxic conditions, leading to rapid accumulation of HIF-1α and HIF-2α, dimerization with HIF-1ß, and transcriptional activation of a large battery of target genes. The increase in ALDH^+^ BCSCs observed after exposure of cells to hypoxia was lost in subclones in which HIF-1α expression was silenced by short hairpin RNA (shRNA), whereas HIF-2α loss-of-function had no effect [3]. Overexpression of HIF-1α in breast cancer is associated with increased patient mortality and HIF target genes play critical roles in angiogenesis, migration, invasion, and metastasis to lymph nodes, lungs, and bone [18-30]. The basal-like breast cancer transcriptional profile is characterized by increased expression of HIF target genes [31]. Here we delineate molecular mechanisms by which HIF-1-dependent activation of TAZ expression and activity induces the BCSC phenotype in response to hypoxia.

## RESULTS

### Hypoxia induces HIF-1-dependent expression of TAZ

Gene expression data from 1,160 human breast cancer specimens in the TCGA database were used to compare levels of TAZ mRNA with the expression of CXCR3, L1CAM, LOX, P4HA1, P4HA2, PDGFB, PLOD1, PLOD2, SLC2A1, and VEGFA mRNA, which are all HIF-regulated in breast cancer cells (Fig. S1A). Statistical analysis revealed that TAZ expression was significantly correlated with 8 out of 10 HIF-1 target genes (Fig. S1B). A HIF metagene signature, based on the combined expression of all 10 HIF-1 target genes, was also correlated with TAZ mRNA expression (Fig. S1C). These data suggest that TAZ mRNA expression may be HIF-regulated in human breast cancers, particularly in basal-like breast cancers.

**Figure 1:**
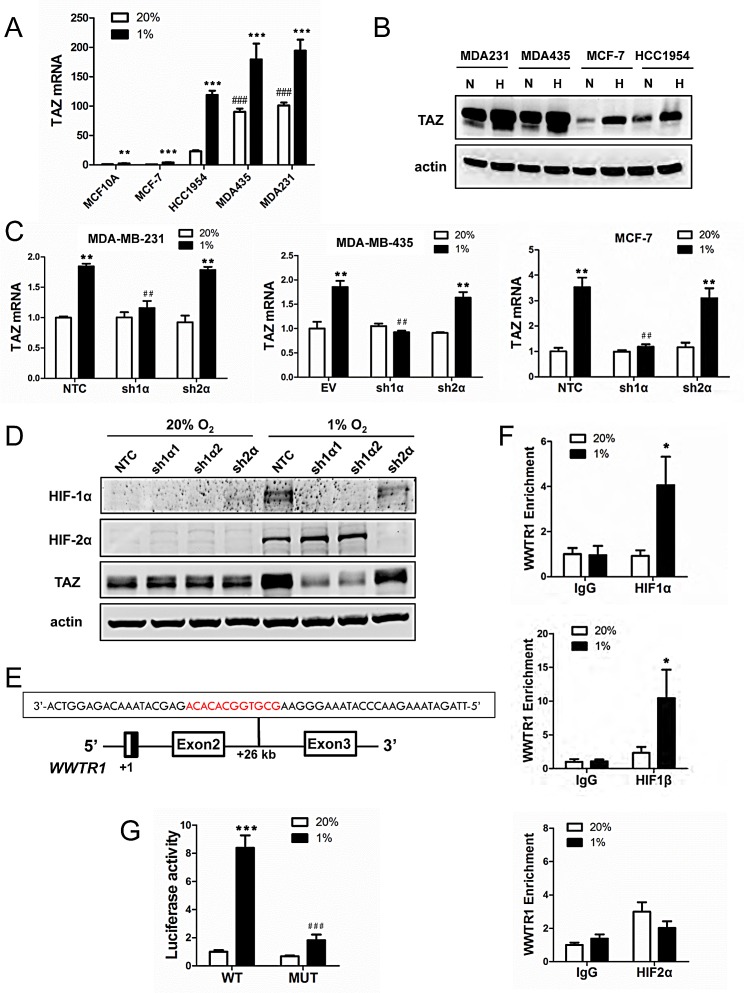
The *WWTR1* gene encoding TAZ is a HIF-1 target gene (A) Reverse transcription and quantitative real-time PCR (RT-qPCR) were performed to quantify TAZ mRNA levels in 5 breast cell lines following exposure to 20% or 1% O_2_ for 24 h. For each sample, the expression of TAZ mRNA was quantified relative to 18*S* rRNA and then normalized to lane 1 (mean ± SEM; n = 3). ^**^P < 0.01, ^***^P < 0.001 versus MCF-10A at 20% O_2_; ^###^*P* < 0.001 versus MCF-10A at 1% O_2_. (B) Immunoblot assays were performed to analyze TAZ protein expression in breast cell lines following exposure to 20% (N) or 1% (H) O_2_ for 48 h. (C) TAZ mRNA expression was analyzed by RT-qPCR in the MDA-MB-231 (left), MDA-MB-435 (middle) and MCF-7 (right) non-targeting control (NTC) or empty vector (EV) subclone and subclones expressing shRNA targeting HIF-1α (sh1α) or HIF-2α (sh2α), which were exposed to 20% or 1% O_2_ for 24 h. Data were normalized to lane 1 in each bar graph (mean ± SEM; n = 3). ** *P* < 0.01 versus NTC or EV at 20% O_2_; ^##^ *P* < 0.01 versus NTC or EV at 1% O_2_. (D) Immunoblot assays were performed to analyze HIF-1α, HIF-2α, TAZ, and actin protein expression using lysates prepared from MDA-MB-231 subclones exposed to 20% or 1% O_2_ for 48 h. (E) The nucleotide sequence (non-coding strand) of a hypoxia response element (HRE; 5′-GCGTG-3′ HIF-1 binding site and 5′-CACA-3′ accessory sequence are shown in red) within intron 2 of the *WWTR1* gene, located 26 kb from the transcription start site, is shown. Exons and intron are not drawn to scale. (F) HCC-1954 cells were exposed to 20% or 1% O_2_ for 16 h and chromatin immunoprecipitation (ChIP) assays were performed using IgG or antibodies against HIF-1α (upper panel), HIF-1β (middle panel), or HIF-2α (lower panel). Primers flanking the HRE were used for qPCR and results were normalized to lane 1 (mean ± SEM; n = 3). ^*^P < 0.05 versus 20% O_2_ (Student's *t* test). (G) The *WWTR1* HRE containing a wild type (WT: 5′-GCGTG-3′) or mutant (MUT; 5′-GAAAG-3′) HIF-1 binding site was inserted into pGL2-Promoter (encoding firefly luciferase) and co-transfected with pSV-Renilla (encoding Renilla luciferase) into HCC-1954 cells, which were incubated at 20% or 1% O_2_ for 24 h. The firefly:Renilla luciferase ratio (Luciferase activity) was normalized to lane 1 (mean ± SEM; n = 3). ^***^P < 0.001 versus WT at 20% O_2_; ^###^*P* < 0.001 versus WT at 1% O_2_ (Student's *t* test).

To determine whether TAZ expression is induced by hypoxia, TAZ mRNA and protein levels were analyzed in immortalized but non-tumorigenic MCF10A mammary epithelial cells, tumorigenic but non-metastatic MCF-7 and HCC-1954 breast cancer cells, and metastatic MDA-MB-231 and MDA-MB-435 breast cancer cells, which were exposed to non-hypoxic (20% O_2_) or hypoxic (1% O_2_) conditions for 24 h. Reverse transcription (RT) and quantitative real-time PCR (qPCR) assays revealed that the expression of TAZ mRNA under non-hypoxic conditions was greatly increased in the two metastatic basal-like lines compared to the other breast cell lines, but expression was significantly induced by hypoxia in all 5 lines (Fig. 1A and Fig. S1D). TAZ protein was also highly expressed in the metastatic lines at 20% O_2_ and its expression was induced by hypoxia in all cell lines assayed (Fig. 1B). To analyze TAZ expression *in vivo*, we injected MDA-MB-231 cells into the mammary fat pad of female severe combined immunodeficiency (SCID) mice. Immunohistochemistry of tumor sections revealed intense HIF-1α staining in perinecrotic (hypoxic) regions, which co-localized with TAZ and with P4HA1, which is a known HIF-1 target gene product (Fig. S1E).

To determine whether HIF-1α or HIF-2α was required for TAZ expression under hypoxic conditions, we analyzed MDA-MB-231 and MCF-7 subclones, which were stably transfected with an expression vector encoding short hairpin RNA (shRNA) targeting HIF-1α (sh1α) or HIF-2α (sh2α) or a non-targeting control shRNA (NTC), and MDA-MB-435 subclones, which were stably transfected with an empty vector (EV) or expression vector encoding shRNA targeting HIF-1α (sh1α) or HIF-2α (sh2α) [21]. Hypoxic induction of TAZ mRNA expression was lost in MDA-MB-231, MDA-MB-435 and MCF-7 cells when HIF-1α (but not HIF-2α) was silenced (Fig. 1C). Hypoxic induction of TAZ protein expression was also abrogated by two different shRNAs (sh1α1 and sh1α2) targeting HIF-1α (Fig. 1D).

In previous analyses of several other HIF-1 target genes, we identified HREs that contained a match to the HIF binding site consensus 5′-RCGTG-3′ followed after 1 to 8 bp by 5′-CACA-3′ [32]. Analysis of the human *WWTR1* gene, which encodes TAZ, revealed the sequence 5′-GCGTGGCACACA-3′ on the antisense strand within intron 2, at a distance of 26 kb 3′ to the transcription start site (Fig. 1E). To determine whether HIF-1 binds at this site, chromatin immunoprecipitation (ChIP) assays were performed in HCC-1954 breast cancer cells, which demonstrated hypoxia-inducible binding of HIF-1α and HIF-1β, but not HIF-2α, at this site (Fig. 1F). To test whether this putative HRE was functional, a 55-bp oligonucleotide spanning the HIF-1 binding site (Fig. 1E) was inserted into the reporter plasmid pGL2-promoter, in which a basal SV40 promoter drives firefly luciferase expression. HCC-1954 cells were co-transfected with pGL2/*WWTR1*-HRE and pSV-Renilla, in which the basal SV40 promoter drives Renilla luciferase expression, and exposed to 20% or 1% O_2_ for 24 h. The ratio of firefly:Renilla luciferase activity increased significantly in hypoxic HCC-1954 cells (Fig. 1G). Mutation of 5′-CGT-3′ to 5′-AAA-3′ in the HIF-1 binding site of the *WWTR1* HRE (underscored in text above) significantly decreased hypoxia-induced luciferase activity (compare MUT to WT in Fig. 1G). Taken together, the data in Fig. 1 and Fig. S1 demonstrate that HIF-1 binds directly to the *WWTR1* gene and activates its transcription in hypoxic breast cancer cells, leading to increased TAZ mRNA and protein expression.

### Expression of TAZ target genes in breast cancer cells is induced by hypoxia

**Figure 2:**
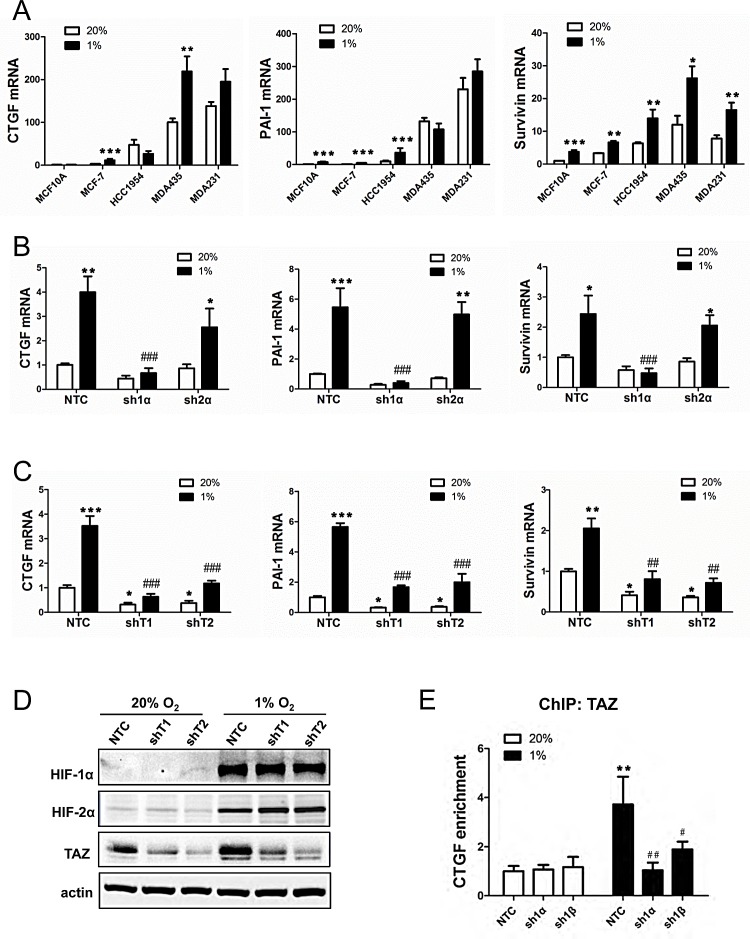
Analysis of TAZ target gene expression (A) RT-qPCR was performed to quantify CTGF, PAI-1 and Survivin mRNA levels in breast cell lines following exposure to 20% or 1% O_2_ for 24 h. For each sample, the expression of CTGF, PAI-1 and Survivin mRNA was quantified relative to 18S rRNA and normalized to lane 1 (mean ± SEM; n = 3). ^*^P < 0.05, ^**^P < 0.01, ^***^P < 0.001 versus 20% O_2_ (two-way ANOVA with Bonferroni post-test). (B-C) MCF-7 subclones expressing NTC, sh1α, sh2α or either of two shRNAs targeting TAZ (shT1, shT2) were exposed to 20% or 1% O_2_ for 24 h and expression of CTGF, PAI-1 and Survivin mRNA was analyzed by RT-qPCR. Data were normalized to lane 1 (mean ± SEM; n = 3). ^*^P < 0.05, ^**^P < 0.01, ^***^P < 0.001 versus NTC at 20% O_2_; ^##^*P* < 0.01, ^###^*P* < 0.001 versus NTC at 1% O_2_. (D) MDA-MB-231 subclones were exposed to 20% or 1% O_2_ for 48 h and immunoblot assays were performed. (*E*) MCF-7 subclones were exposed to 20% or 1% O_2_ for 16 h. Chromatin was immunoprecipitated with anti-TAZ antibody and analyzed by qPCR with primers spanning the *CTGF* promoter (mean ± SEM, n =3). ^**^P < 0.01 versus NTC at 20% O_2_; ^#^*P* < 0.05, ^##^ *P* < 0.01 versus NTC at 1% O_2_.

CTGF, PAI-1, and Survivin mRNA levels under non-hypoxic conditions were highest in the metastatic breast cancer lines (Fig. 2A), which was similar to the pattern of TAZ mRNA expression (Fig. 1A). In response to hypoxia, CTGF mRNA expression increased in MCF-7 and MDA-MB-435 cells, PAI-1 mRNA expression increased in MCF-10A, MCF-7 and HCC-1954 cells, whereas Survivin mRNA expression increased in all five breast cell lines (Fig. 2A). Hypoxic induction of CTGF, PAI-1, and survivin mRNA expression was selectively lost when expression of HIF-1α (but not HIF-2α) was abrogated by shRNA (Fig. 2B). In contrast, in MDA-MB-231 subclones stably transduced with expression vector encoding either of two independent shRNAs targeting TAZ (shT1 and shT2), knockdown of TAZ expression decreased CTGF, PAI-1, and Survivin mRNA expression at both 20% and 1% O_2_ (Fig. 2C). Immunoblot assays confirmed effective knockdown of TAZ protein expression, which did not affect HIF-1α or HIF-2α protein levels (Fig. 2D). ChIP assays revealed that hypoxia increased binding of TAZ to the *CTGF* promoter, which was dependent on the expression of HIF-1α and HIF-1ß (Fig. 2E). These data suggest that HIF-1 indirectly regulates TAZ target genes by increasing TAZ expression.

### Hypoxia induces HIF-1α-dependent TAZ nuclear localization

**Figure 3:**
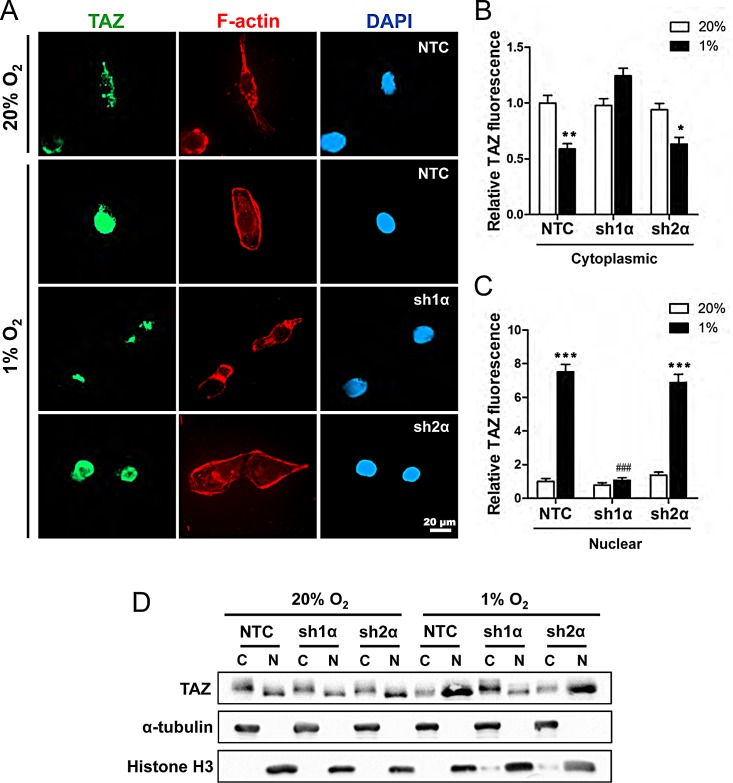
Analysis of TAZ subcellular localization (A) MDA-MB-231 subclones (NTC, sh1α, and sh2α) were exposed to 20% or 1% O_2_ for 48 h and stained with anti-TAZ antibody (green), Alexa Fluor 568-conjugated phalloidin to detect cytosolic F-actin (red), and DAPI to detect nuclear DNA (blue). (B-C) Image analysis was performed to determine the cytoplasmic (B) or nuclear (C) TAZ fluorescence intensity per cell normalized to lane 1 (mean ± SEM; n = 50 cells). ^*^P < 0.05, ^**^P < 0.01, ^***^P < 0.001 versus NTC at 20% O_2_; ^###^*P* < 0.001 versus NTC at 1% O_2_ (two-way ANOVA with Bonferroni post-test). (D) Immunoblot assays of TAZ, histone H3, and α-tubulin protein in cytosolic (C) and nuclear (N) lysates prepared from MDA-MB-231 subclones (NTC, sh1α, sh2α) exposed to 20% or 1% O_2_ for 48 h.

Transcriptional regulation of TAZ by HIF-1 is surprising because most studies to date have focused on the regulation of TAZ subcellular localization by LATS1 and LATS2, which inhibit the nuclear localization of TAZ [7]. To determine whether hypoxia affects the subcellular localization of TAZ, MDA-MB-231 subclones were incubated at 20% or 1% O_2_ for 48 h and stained with anti-TAZ antibody, Alexa 568-conjugated phalloidin (to detect F-actin in the cytoplasm), and DAPI (to stain nuclear DNA) (Fig. 3A). TAZ was localized primarily in the cytoplasm of non-hypoxic cells (Fig. 3B). In NTC and sh2α cells, but not in sh1α cells, exposure to hypoxia increased the expression of TAZ, which was localized primarily to nuclei (Fig. 3C), with decreased levels in the cytoplasm (Fig. 3B). We also prepared cytoplasmic and nuclear extracts, which were validated by immunoblot assays for α-tubulin and histone H3, respectively, and confirmed that hypoxia induced TAZ nuclear localization in a HIF-1α-dependent and HIF-2α-independent manner (Fig. 3D).

### Hypoxia induces proteasomal degradation of LATS2 that is dependent on HIF-1α and SIAH1

**Figure 4:**
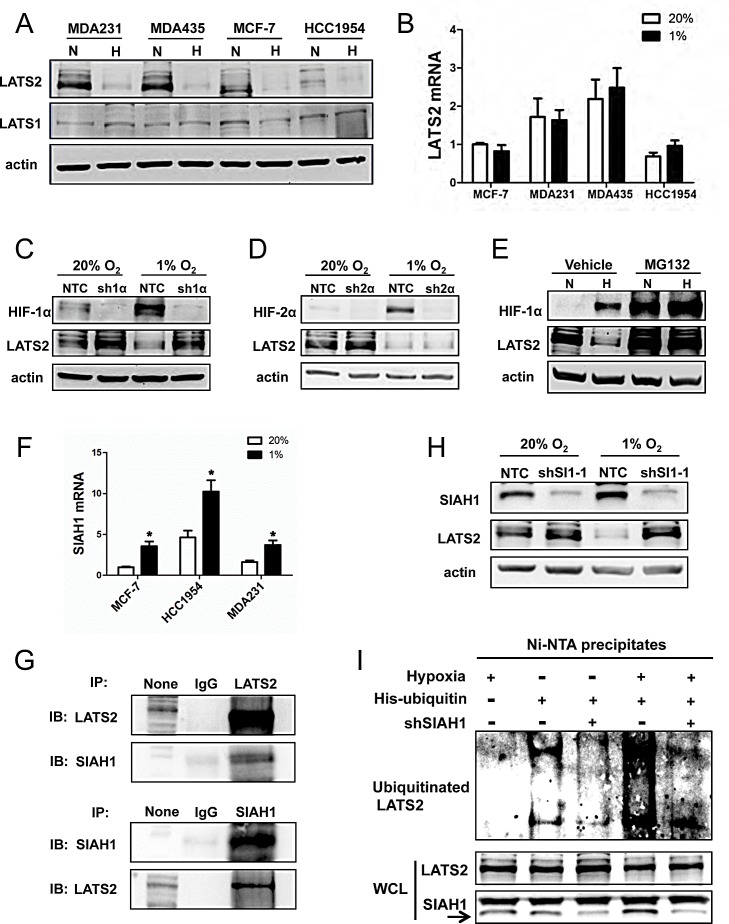
Analysis of LATS2 expression (A) Immunoblot assays were performed to analyze actin, LATS1, and LATS2 protein levels in 4 breast cancer cell lines following exposure to 20% (N) or 1% (H) O_2_ for 48 h. (B) RT-qPCR was performed to quantify LATS2 mRNA levels in 4 breast cancer cell lines following exposure to 20% or 1% O_2_ for 24 h. For each sample, the expression of LATS2 mRNA was quantified relative to 18S rRNA and then normalized to lane 1 (mean ± SEM; n = 3). (C-D) Immunoblot assays were performed using antibodies against HIF-1α (C), HIF-2α (D), LATS2, and actin with whole cell lysates (WCLs) prepared from MDA-MB-231 subclones sh1α (C), sh2α (D) and NTC (C and D), which were exposed to 20% or 1% O_2_ for 48 h. (E) MDA-MB-231 cells were exposed to 20% O_2_ (N) or 1% O_2_ (H) for 48 h, with 10 μM MG132 or vehicle added for the last 6 h, and immunoblot assays were performed using antibodies against HIF-1α, LATS2, and actin. (F) RT-qPCR was performed to determine SIAH1 mRNA levels in breast cell lines following exposure to 20% or 1% O_2_ for 24 h. For each sample, the expression of SIAH1 mRNA was quantified relative to 18S rRNA and then normalized to the result obtained from MCF-7 cells at 20% O_2_ (mean ± SEM; n = 3). ^*^P < 0.05 versus 20% O_2_ (Student's *t* test). (G) Immunoprecipitation (IP) was performed using IgG, anti-LATS2 (upper panels) or anti-SIAH1 (lower panel) antibody and WCL from MDA-MB-231 cells exposed to 1% O_2_ for 48 h with 10 μM MG132 added for the last 6 h. Immunoblot (IB) assays were performed with antibodies against LATS2 or SIAH1. None indicates aliquot of WCL prior to IP. (H) MDA-MB-231 cells were stably transduced with lentiviral vectors encoding NTC or shSIAH1 (shSI1) and exposed to 20% or 1% O_2_ for 48 h. Immunoblot assays were performed with antibodies against SIAH1, LATS2, and actin. (I) MDA-MB-231 cells were co-transfected with vectors encoding His-tagged ubiquitin and SIAH1 shRNA (shSIAH1^+^) or non-targeting control shRNA (shSIAH1^−^) and exposed to 1% O_2_ for 48 h with 10 μM MG132 added for the last 6 h. Total ubiquitinated proteins were precipitated from WCLs by Ni-NTA beads and subject to immunoblot assay with anti-LATS2 antibodies (upper panel). Aliquots of WCL reserved prior to immunoprecipitation were assayed with antibodies against LATS2 and SIAH1 (lower panels).

We next analyzed the expression of LATS1 and LATS2, which negatively regulate TAZ nuclear localization [7]. Whereas LATS1 protein was constitutively expressed at low levels, LATS2 protein was highly expressed under non-hypoxic conditions and expression was dramatically decreased under hypoxic conditions in the four breast cancer cell lines that were analyzed (Fig. 4A). In contrast to LATS2 protein, LATS2 mRNA levels were not O_2_-regulated (Fig. 4B). The reduction in LATS2 protein levels in response to hypoxia was observed in NTC and sh2α cells, but not in sh1α cells (Fig. 4, C and D). Treatment of cells with the proteasome inhibitor MG132 blocked the degradation of HIF-1α in non-hypoxic cells and LATS2 in hypoxic cells (Fig. 4E).

The proteasome-dependent degradation of LATS2 suggested that it might be subject to ubiquitination. SIAH1 and SIAH2 are E3 ubiquitin-protein ligases that are induced by hypoxia [33]. Analysis of gene expression data from human breast cancers revealed that SIAH1, but not SIAH2, mRNA expression showed a significant positive correlation with the expression of multiple HIF target genes (Fig. S2A). The expression of mRNA encoding SIAH1, but not SIAH2, was also increased under hypoxic conditions in breast cancer cell lines (Fig. 4F and S2B). Next, MDA-MB-231 cells were exposed to 1% O_2_ for 48 h with MG132 treatment for the last 6 h. Co-immunoprecipitation assays revealed physical interaction of LATS2 and SIAH1 (Fig. 4G). Knockdown of SIAH1 expression in MDA-MB-231 cells (shSI1-1 subclone) blocked the hypoxia-induced degradation of LATS2 (Fig. 4H). To analyze the effect of SIAH1 on LATS2 ubiquitination, MDA-MB-231 cells were co-transfected with vectors encoding His-tagged ubiquitin and shSI1-1 or NTC. The shSI1-1 vector, but not the NTC vector, markedly reduced levels of SIAH1 protein (Fig. 4I, bottom panel). Cells were exposed to hypoxia and treated with the proteasome inhibitor MG132 to block LATS2 degradation. The polyubiquitination of LATS2 was dramatically inhibited in SIAH1 knockdown cells as compared with NTC cells (Fig. 4I, top panel), whereas total LATS2 levels were similar (Fig. 4I, bottom panel).

### Hypoxia induces SIAH1 expression in a HIF-1-dependent manner

**Figure 5:**
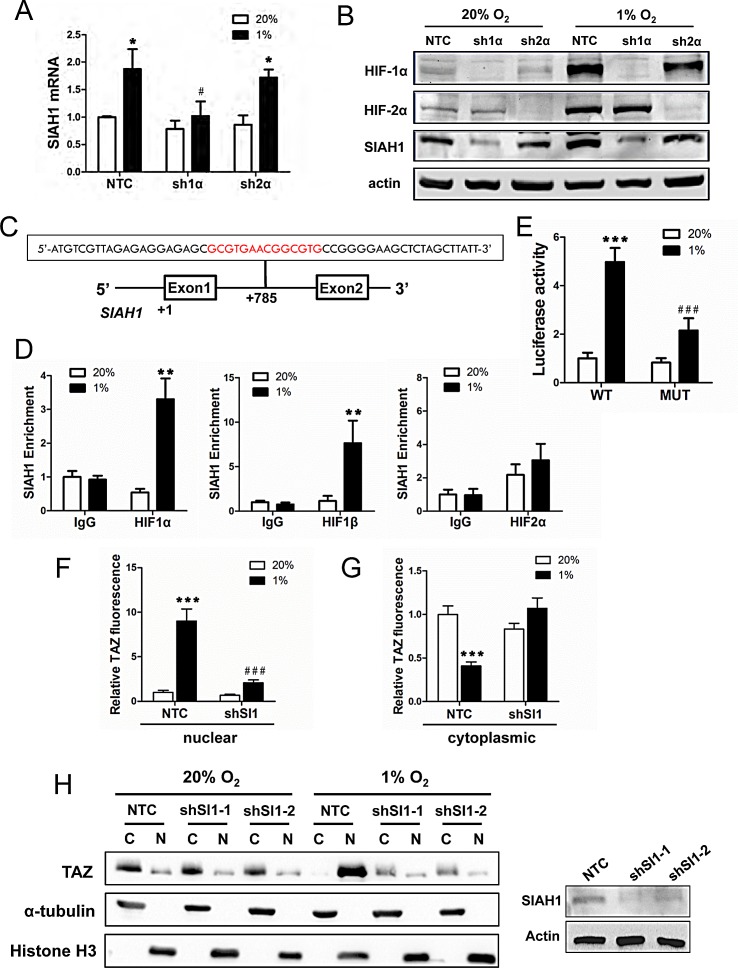
*SIAH1* is a HIF-1 target gene product that regulates TAZ nuclear localization (A) SIAH1 mRNA levels were analyzed by RT-qPCR in MDA-MB-231 subclones (NTC, sh1α, sh2α), which were exposed to 20% or 1% O_2_ for 24 h. The data were normalized to lane 1 (mean ± SEM; n = 3). ^*^P < 0.05 versus NTC at 20% O_2_; ^#^*P* < 0.05 versus NTC at 1% O_2_. (B) Immunoblot assays were performed using antibodies against HIF-1α, HIF-2α, SIAH1, and actin with WCLs prepared from MDA-MB-231 subclones (NTC, sh1α, sh2α) exposed to 20% or 1% O_2_ for 48 h. (C) The nucleotide sequence of a candidate HRE (two copies of HIF-1 binding site 5′-GCGTG-3′ are shown in red) within intron 1 of the human *SIAH1* gene, located 785 bp 3′ to the transcription start site, is shown. Exons and intron are not drawn to scale. (D) MCF-7 cells were exposed to 20% or 1% O_2_ for 16 h and ChIP assays were performed using IgG or antibodies against HIF-1α, HIF-2α or HIF-1β. Primers flanking the HRE were used for qPCR and results were normalized to lane 1 (mean ± SEM; n = 3). ^**^P < 0.01 versus 20% O_2_ (Student's *t* test). (E) The *SIAH1* HRE containing wild type (WT: 5′-GCGTGAACGGCGTG-3′) or mutant (MUT; 5′-GAAAGAACGGAAAG-3′) HIF-1 binding sites was inserted into pGL2-Promoter (encoding firefly luciferase) and co-transfected with pSV-Renilla (encoding Renilla luciferase) into MCF-7 cells, which were incubated at 20% or 1% O_2_ for 24 h. The firefly:Renilla luciferase ratio (Luciferase activity) was normalized to lane 1 (mean ± SEM; n = 3). ^***^P < 0.001 versus WT at 20% O_2_; ^###^*P* < 0.001 versus WT at 1% O_2_ (Student's *t* test). (F-G) NTC or shSI-1 subclones were exposed to 20% or 1% O_2_, subjected to immunofluorescent assays, and image analysis was performed to determine the nuclear (F) or cytoplasmic (G) TAZ fluorescence intensity per cell normalized to lane 1 (mean ± SEM; n = 50 cells). ^***^P < 0.001 versus NTC at 20% O_2_; ^###^*P* < 0.001 versus NTC at 1% O_2_ (two-way ANOVA with Bonferroni post-test). (H) Left panel: Immunoblot assays of TAZ, histone H3, and α-tubulin protein were performed using cytosolic (C) and nuclear (N) lysates prepared from MDA-MB-231 subclones (NTC, shSI1-1, shSI1-2) that were exposed to 20% or 1% O_2_ for 48 h. Right panel: Immunoblot assays were performed using antibodies against SIAH1 and actin with WCLs prepared from MDA-MB-231 NTC and SIAH1 knockdown subclones.

Having found that SIAH1, but not SIAH2, mRNA increased under hypoxia in breast cancer cell lines, we next investigated whether *SIAH1* gene expression is HIF-regulated. Analysis of MDA-MB-231 subclones revealed that expression of SIAH1 mRNA (Fig. 5A) and protein (Fig. 5B) was induced by hypoxia in NTC and sh2α cells but not in sh1α cells. Analysis of the human *SIAH1* gene revealed the sequence 5′-GCGTGAACGGCGTG-3′, containing two potential HIF binding sites (in bold), on the sense strand within intron 1, at a distance of 785 bp 3′ to the transcription start site (Fig. 5 C). To determine whether HIFs bind at this site, ChIP assays were performed in MCF-7 cells, which demonstrated hypoxia-inducible binding of HIF-1α and HIF-1β, but not HIF-2α, at this site (Fig. 5 D). Similar results were obtained using HCC-1954 cells (Fig. S2, C-E). To test whether this putative HRE was functional, a 55-bp oligonucleotide spanning the site (Fig. 5C) was inserted into the reporter plasmid pGL2-promoter. MCF-7 cells were co-transfected with pGL2/*SIAH1*-HRE and pSV-Renilla and exposed to 20% or 1% O_2_ for 24 h. The ratio of firefly:Renilla luciferase activity increased significantly in hypoxic MCF-7 cells (Fig. 5E). Mutation of the two HIF-1 binding sites (5′-CGT-3′ to 5′-AAA-3′) in the SIAH1 HRE (underscored in text above) significantly decreased hypoxia-induced luciferase activity (compare MUT to WT in Fig. 5E).

### Nuclear localization of TAZ is dependent on SIAH1

To determine whether hypoxia-induced SIAH1 expression affects the subcellular localization of TAZ, MDA-MB-231 subclones were incubated at 20% or 1% O_2_ for 48 h and stained with anti-TAZ antibody, as well as Alexa 568-conjugated phalloidin and DAPI (Fig. S3). TAZ was localized primarily in the cytoplasm of cells exposed to 20% O_2_ whereas in NTC cells, but not in shSI1-1 cells, exposure to hypoxia increased the expression of TAZ, which was localized primarily to nuclei, with decreased levels in the cytoplasm (Fig. 5, F and G). Similar results were obtained by immunoblot assays of nuclear and cytoplasmic extracts from shSI1-1 or shSI1-2 cells (Fig. 5, H and I). Taken together, the data in Fig. 4 and Fig. 5 indicate that HIF-1 regulates the nuclear localization of TAZ by triggering the SIAH1-dependent proteasomal degradation of LATS2 in hypoxic breast cancer cells.

### HIF-1α, SIAH1, and TAZ regulate the breast cancer stem cell phenotype

**Figure 6:**
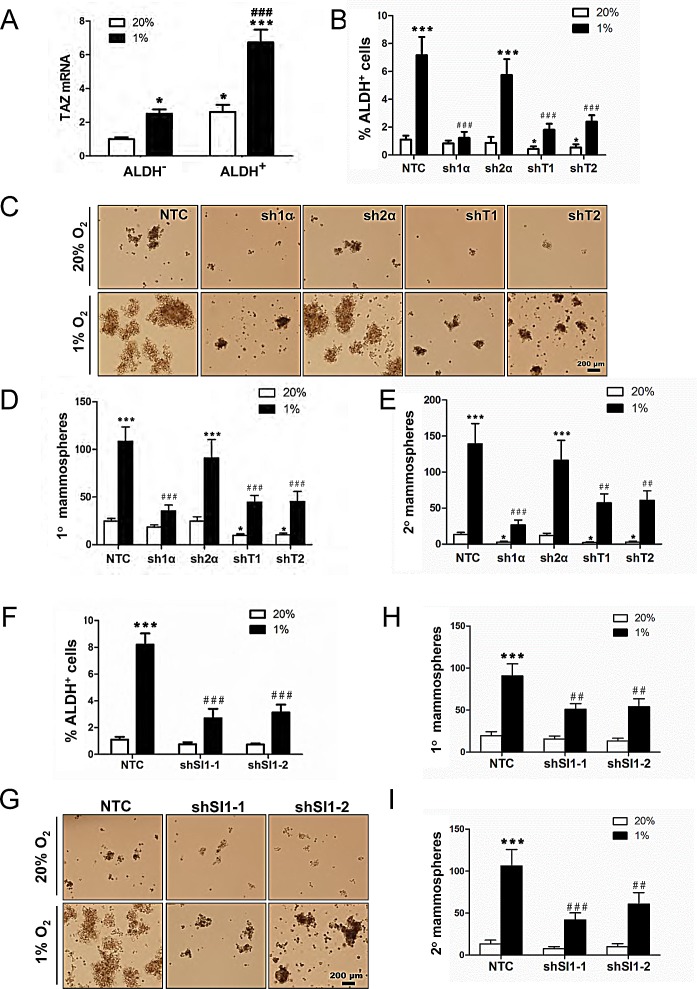
HIF-1α, TAZ, and SIAH1 regulate the breast cancer stem cell phenotype (A) MDA-MB-231 cells were exposed to 20% or 1% O_2_ for 72 h, cells were sorted into aldehyde dehydrogenase^+^ (ALDH^+^) and ALDH^−^ populations by FACS, and TAZ mRNA expression was determined by RT-qPCR and normalized to lane 1 (mean ± SEM; n = 6). ^*^P < 0.05 versus ALDH^−^ at 20% O_2_; ^***^P < 0.001 versus ALDH^+^ at 20% O_2_; ^###^*P* < 0.001 versus ALDH^−^ at 1% O_2_. (B) MDA-MB-231 subclones (NTC, sh1α, sh2α, shT1, shT2) were exposed to 20% or 1% O_2_ for 72 h and the percentage of ALDH^+^ cells was determined by flow cytometry (mean ± SEM; n = 3). ^*^P < 0.05, ^***^P < 0.001 versus NTC at 20% O_2_; ^###^*P* < 0.001 versus NTC at 1% O_2_. (C-E) MDA-MB-231 subclones were exposed to 20% or 1% O_2_ for 72 h. Cells were seeded at a density of 5,000 cells/mL, cultured for 7 d (C), and the number of primary mammospheres ≥ 70 μm in diameter was counted. The mammospheres were dissociated and seeded at a density of 2,000 cells/mL, cultured for 7 d, and secondary mammospheres were counted. The number of primary (D) and secondary (E) mammospheres per 1000 cells initially seeded was calculated (mean ± SEM; n = 3). ^*^P < 0.05, ^***^P < 0.001 versus NTC at 20% O_2_; ^##^*P* < 0.01, ^###^*P* < 0.001 versus NTC at 1% O_2_. (F) MDA-MB-231 subclones (NTC, shSI1-1, shSI1-2) were exposed to 20% or 1% O_2_ for 72 h and the percentage of ALDH^+^ cells was determined by Aldefluor assay and flow cytometry (mean ± SEM; n = 3). ^***^P < 0.001 versus NTC at 20% O_2_; ^###^*P* < 0.001 versus NTC at 1% O_2_. (G-I) MDA-MB-231 subclones (NTC, shSI1-1, shSI1-2) were exposed to 20% or 1% O_2_ for 72 h. Cells were seeded at a density of 5,000 cells/mL, cultured for 7 d (G) and the number of primary mammospheres ≥ 70 μm in diameter was counted. The mammospheres were enzymatically dissociated, and the resulting single cells were seeded at a density of 2,000 cells/mL, cultured for 7 d, and secondary mammospheres were counted. The number of primary (H) and secondary (I) mammospheres per 1000 cells initially seeded was calculated (mean ± SEM; n = 3). ^***^P < 0.001 versus NTC at 20% O_2_; ^##^*P* < 0.01, ^###^*P* < 0.001 versus NTC at 1% O_2_.

TAZ positively regulates BCSCs [10] and the enrichment of ALDH^+^ BCSCs under hypoxic conditions is HIF-1α-dependent [3]. MDA-MB-231 cells were exposed to 20% or 1% O_2_ for 72 h and the Aldefluor assay was performed followed by FACS to collect the ALDH^+^ population, which is highly enriched for BCSCs, and the ALDH^−^ population, which is depleted of BCSCs. TAZ mRNA levels were significantly increased in ALDH^+^ as compared to ALDH^−^ cells, and hypoxia induced TAZ expression in both cell populations (Fig. 6A). Analysis of MDA-MB-231 subclones revealed that hypoxia markedly increased the percentage of ALDH^+^ NTC and sh2α cells, whereas the hypoxic induction of BCSCs was abrogated in the sh1α subclone; in contrast, ALDH^+^ BCSCs were significantly decreased in the shT1 and shT2 subclones at both 20% and 1% O_2_ (Fig. 6B).

To analyze whether HIF-1α or TAZ is required for BCSC self-renewal, mammosphere formation assays were performed. MDA-MB-231 subclones were exposed to 20% or 1% O_2_ for 72 h in adherent culture, then the cells were cultured on ultra-low attachment plates for 7 d, and the resulting primary mammospheres were counted. Hypoxia increased primary mammosphere formation by NTC or sh2α cells, but not by sh1α, cells (Fig. 6, C and D). The primary mammospheres were dissociated, replated on ultra-low attachment plates, and the number of secondary mammospheres was counted 7 d later. Secondary mammosphere formation was also markedly increased following exposure of NTC or sh2α cells to hypoxia, whereas hypoxia-induced mammosphere formation was significantly impaired when sh1α, shT1, or shT2 cells were used; non-hypoxic cultures of these cells also showed impaired secondary mammosphere formation compared to NTC or sh2α cells (Fig. 6E).

If hypoxic induction of the BCSC phenotype is due in part to SIAH1-dependent degradation of LATS2, then SIAH1 knockdown should also reduce the BCSC population. Analysis of MDA-MB-231 subclones revealed that hypoxia markedly increased the percentage of ALDH^+^ NTC cells, whereas the hypoxic induction of BCSCs was significantly decreased in two independent SIAH1 knockdown (shSI1-1 and shSI1-2) subclones (Fig. 6F). Hypoxia increased primary mammosphere formation when NTC cells were used, but not when SIAH1 knockdown subclones were used (Fig. 6, G and H). Hypoxia-induced secondary mammosphere formation was also significantly impaired when SIAH1 knockdown subclones were used (Fig. 6I).

### LATS2 knockdown induces breast cancer stem cell phenotype and TAZ nuclear localization

**Figure 7:**
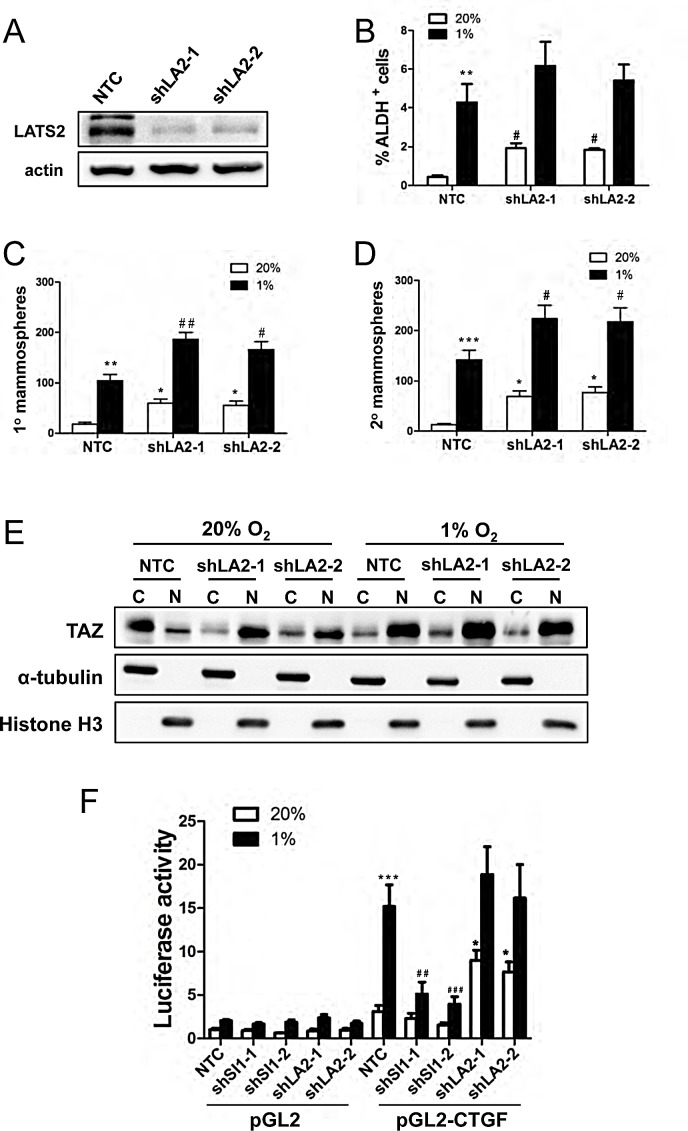
LATS2 knockdown induces breast cancer stem cell phenotype and TAZ nuclear localization (A) Immunoblot assays were performed using antibodies against LATS2 and actin with cell extracts prepared from MDA-MB-231 subclones (NTC, shLA2-1, shLA2-2). (B) MDA-MB-231 subclones (NTC, shLA2-1, shLA2-2) were exposed to 20% or 1% O_2_ for 72 h and the percentage of ALDH^+^ cells was determined by Aldefluor assay and flow cytometry (mean ± SEM; n = 3). ^**^P < 0.01 versus NTC at 20% O_2_; ^#^*P* < 0.05 versus NTC at 1% O_2_. (C-D) MDA-MB-231 subclones (NTC, shLA2-1, shLA2-2) were exposed to 20% or 1% O_2_ for 72 h. The number of primary (C) and secondary (D) mammospheres per 1000 cells initially seeded was determined (mean ± SEM; n = 4). ^*^P < 0.05, ^**^P < 0.01, ^***^P < 0.001 versus NTC at 20% O_2_; ^#^*P* < 0.05, ^##^*P* < 0.01 versus NTC at 1% O_2_. (E) Immunoblot assays of TAZ, histone H3, and α-tubulin protein were performed using cytosolic (C) and nuclear (N) lysates prepared from MDA-MB-231 subclones (NTC, shLA2-1, shLA2-2) that were exposed to 20% or 1% O_2_ for 48 h. (F) MCF-7 cells were transfected with pGL2 or pGL2-CTGF, pSV-Renilla, and an expression vector encoding a non-targeting control shRNA (NTC) or two independent shRNAs targeting LATS2 or SIAH1, and exposed to 20% or 1% O2 for 24 h. The luciferase activity was normalized to lane 1 (mean ± SEM, n = 4). ^*^P < 0.05, ^***^P < 0.001 versus NTC at 20% O_2_; ^##^ *P* < 0.01, ^###^ *P* < 0.001 versus NTC at 1% O_2_.

To investigate whether LATS2 loss of function was sufficient to induce the BCSC phenotype, we generated two independent LATS2 knockdown subclones of MDA-MB-231 (Fig. 7A). LATS2 knockdown increased the percentage of ALDH^+^ cells (Fig. 7B) as well as primary and secondary mammosphere formation (Fig. 7, C and D). LATS2 knockdown also increased the nuclear localization of TAZ at 20% O_2_ (Fig. 7E). We next investigated whether SIAH1 or LATS2 knockdown affected TAZ transcriptional activity. A 247-bp *CTGF* gene promoter fragment, which contains three copies of the TAZ/TEAD-binding site sequence (5′-GGAATG-3′) and no match to the HIF-1 binding site consensus sequence (5′-RCGTG-3′), was inserted into pGL2-Basic (pGL2-CTGF). MCF-7 cells were co-transfected with the following plasmids: pGL2-CTGF or pGL2; pSV-Renilla; and an expression vector encoding a non-targeting control shRNA (NTC) or either of two independent shRNAs targeting SIAH1 or LATS2. The ratio of pGL2-CTGF:pSV-Renilla activity was increased in response to hypoxia in NTC cells, whereas knockdown of SIAH1 significantly impaired the induction of luciferase activity under hypoxic conditions. Knockdown of LATS2 increased luciferase activity under non-hypoxic conditions (Fig. 7F). Thus, TAZ transcriptional activity is repressed by LATS2 under non-hypoxic conditions and de-repressed under hypoxic conditions in a SIAH1-dependent manner.

### HIF and TAZ activity affect tumor formation and patient mortality

**Figure 8:**
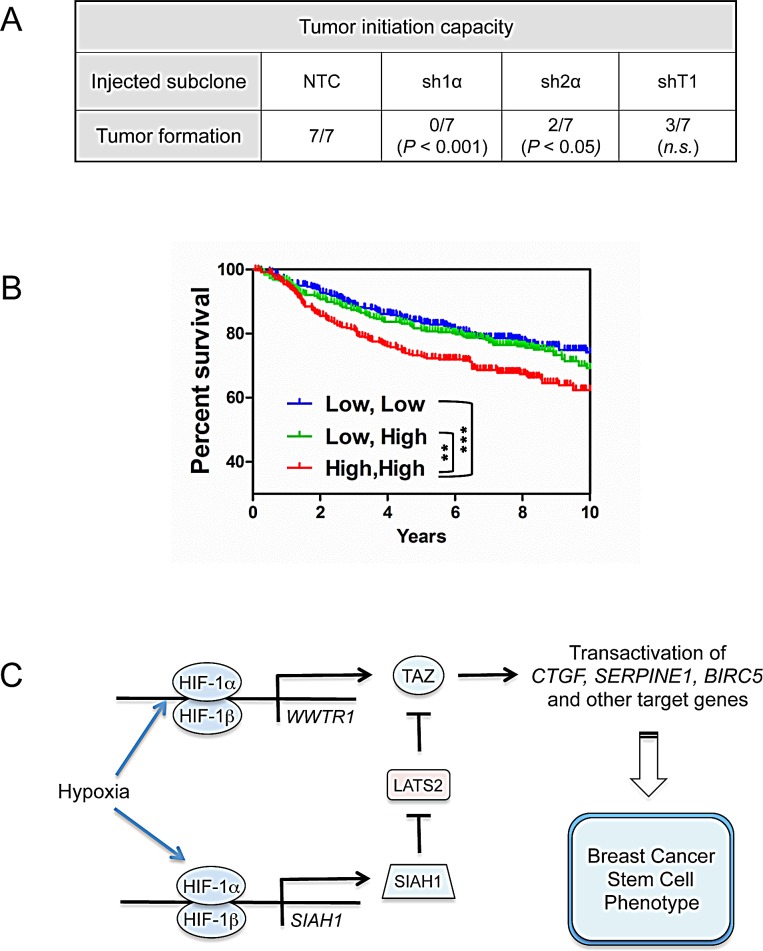
Effect of HIF and TAZ activity on tumorigenicity and patient survival (A) To analyze the tumor-initiating capacity of the NTC, sh1α, sh2α, and shT1 subclones, 1000 cells were injected into the mammary fat pad of immunodeficient female mice. The table reports the number of mice developing palpable tumors after 71 d. The Chi-square test was performed to determine statistical significance versus NTC; n.s., not significant. (B) Kaplan–Meier analysis of disease-specific survival for 1,098 breast cancer patients stratified by TAZ and HIF signatures in the primary tumor. High, High (red): patients with both TAZ and HIF signatures greater than the median (N = 356). Low, High (green): patients with TAZ or HIF signature greater than the median and the other signature less than the median (N = 382). Low, Low (blue): patients with both signatures less than the median (N = 360). The Wilcoxon rank sum test was used to compare survival curves (**P < 0.01, ***P < 0.001). (C) HIF-1 activates transcription of the *WWTR1* and *SIAH1* genes to stimulate TAZ expression and nuclear localization, respectively, and induce the breast cancer stem cell phenotype in response to hypoxia.

To investigate whether HIFs and TAZ regulate the tumor-initiating potential of breast cancer cells *in vivo*, we injected 1000 NTC, sh1α, sh2α, or shT1 cells into the mammary fat pad of female SCID mice. Whereas NTC cells formed tumors in the all of the injected mice by 71 d after injection, HIF-1α knockdown cells showed a complete loss of tumor-initiating ability and knockdown of TAZ or HIF-2α impaired, but did not eliminate, tumor formation (Fig. 8A). In contrast, both HIF [21] and TAZ [10] knockdown subclones have previously been shown to initiate tumors as efficiently as control MDA-MB-231 subclones when > 10^4^ cells were injected. Taken together, these results are consistent with the hypothesis that tumor initiation is dependent on HIF and TAZ activity.

To determine the clinical relevance of HIF-1α and TAZ expression in breast cancer, survival data were analyzed by stratifying patients according to the expression of HIF- and TAZ-regulated genes in the primary tumor. Kaplan-Meier plots revealed that breast cancers with high expression (above the median) of both HIF and TAZ target genes were associated with significantly decreased patient survival over 10 years as compared to cancers with low expression of one or both signatures (Fig. 8B).

## DISCUSSION

The median *P*O_2_ in advanced breast cancers is 10 mmHg (~1.5% O_2_), as compared to 65 mmHg in normal breast tissue, and those cancers below the median *P*O_2_ are at increased risk of metastasis and mortality [34]. Intratumoral hypoxia induces the expression of HIF-1α, which is associated with increased risk of metastasis, relapse, and mortality in multiple clinical studies involving thousands of breast cancer patients [35-44]. Only BCSCs possess the ability to form metastatic and recurrent tumors [45]. The observation that hypoxia increased the ALDH^+^ population in a HIF-1α-dependent manner [3] provided a link between increased HIF-1α levels and BCSCs, but the mechanism by which HIF-1α stimulates the BCSC phenotype was not delineated.

In this study, we have demonstrated that HIF-1 stimulates both the expression and activity of TAZ, a transcriptional co-activator that is required for maintenance of BCSCs [10]. HIF-1α binds to an HRE in the *WWTR1* gene and activates its transcription, leading to increased TAZ mRNA and protein expression; in addition, HIF-1α binds to an HRE in the *SIAH1* gene and activates its transcription, leading to LATS2 ubiquitination and degradation, which increases TAZ nuclear localization (Fig. 8C). While this work was in progress, HIF-1α and TAZ interaction in breast cancer bone metastases was reported and shown to stimulate HIF-1 transcriptional activity [46], suggesting reciprocal positive interactions between HIF-1 and TAZ. However, it is the HIF-1-mediated increase in the expression, nuclear localization, and transcriptional activity of TAZ that provides a novel molecular mechanism by which intratumoral hypoxia induces the BCSC phenotype. The demonstration that HIF-1 mediates increased expression of TAZ and its positive regulator SIAH1 is conceptually similar to our finding that HIFs mediate the expression of Rho Kinase 1 and its positive regulator Rho A to stimulate the motility of hypoxic breast cancer cells [19].

HIF-1α, but not HIF-2α, was required for the hypoxia-induced expression of TAZ and SIAH1, which was consistent with the finding that HIF-1α, but not HIF-2α, was required for hypoxic induction of the BCSC phenotype as measured by the Aldefluor and mammosphere formation assays. However, HIF-2α-deficient cells showed reduced tumorigenicity, which suggests that HIF-2α may be required for aspects of the BCSC phenotype that are not captured by the *in vitro* assays.

Analysis of the TCGA human breast cancer database revealed that the combination of increased HIF and TAZ target gene expression was associated with a significantly increased risk of patient mortality as compared to increased expression of either HIF or TAZ target genes. This finding is consistent with the established role of HIFs in regulating other key pathways involved in breast cancer metastasis. It also may reflect the involvement of other factors in the regulation of TAZ activity. In this regard, it should be noted that hypoxia induced HIF-1-dependent TAZ expression in all breast cancer cell lines, whereas under non-hypoxic conditions, TAZ levels were increased in metastatic breast cancer cells, relative to non-metastatic cells, through a HIF-independent mechanism.

The administration of anti-angiogenic agents to tumor-bearing mice has been shown to increase intratumoral hypoxia and thereby increase the percentage of BCSCs [3]. Our data implicate HIF-1-dependent induction of TAZ transcriptional activity in hypoxia-induced BCSC enrichment and suggest that co-administration of a HIF-1 inhibitor may be necessary to block the counter-therapeutic effects of anti-angiogenic agents.

The basal-like molecular subclass of human breast cancers is characterized by increased expression of HIF target genes and largely overlaps with the triple-negative clinical subclass of breast cancers that lack expression of the estrogen receptor, progesterone receptor, and HER2 [31]. These cancers are currently treated with cytotoxic chemotherapy that results in a durable response in less than 20% of patients. In orthotopic mouse models of triple negative breast cancer, drugs that block HIF-1α accumulation inhibit primary tumor growth and vascularization, local tissue invasion, regional lymph node metastasis, and lung metastasis, as well as reducing the percentage of BCSCs in the primary tumor [20, 21, 47], suggesting that co-administration of a HIF-1 inhibitor with chemotherapy may improve outcome in this patient population.

## METHODS

### Statistical analysis of microarray data

Gene expression data from the Breast Invasive Carcinoma dataset (TCGA) were obtained from https://genome-cancer.ucsc.edu. Pearson's correlation coefficient was used to determine *P* values for co-expression.

### Cell culture

Breast cell lines MCF-10A, MDA-MB-231, MDA-MB-435 and MCF-7 were cultured as previously described [19]. HCC1954 cells were cultured in RPMI-1640 supplemented with 10% fetal bovine serum and 1% penicillin-streptomycin. All cells were maintained at 37°C in a 5% CO_2_/95% air incubator. For hypoxic exposure, cells were placed in a modular incubator chamber (Billups–Rothenberg) and flushed with a 1% O_2_/5% CO_2_/94% N_2_ gas mixture. MG132 was purchased from Calbiochem.

### ShRNA, lentiviruses, and transduction

Vectors encoding shRNA targeting HIF-1α (sh1α1) and HIF-2α were previously described [21]. The shRNA vectors encoding NTC and sh1α2 were purchased from Sigma. pLKO.1-puro lentiviral vectors encoding shRNA targeting TAZ and SIAH1 were purchased from Sigma-Aldrich: shT1 (Clone ID: NM_015472.3-706s1c1); shT2 (Clone ID: NM_015472.3-1657s21c1); shSI1-1 (Clone ID: NM_003031.3-563s21c1); shSI1-2 (Clone ID: NM_003031.3-826s21c1); shLA2-1 (Clone ID: NM_014572.x-3750s1c1) and shLA2-2 (Clone ID: NM_014572.x-2162s1c1). Lentiviral vectors were co-transfected with plasmid pCMV-dR8.91 and a plasmid encoding vesicular stomatitis virus G protein into 293T cells using Lipofectamine 2000 (Invitrogen). Medium containing viral particles was collected 48 h after transfection and passed through a 0.45-μM filter. MDA-MB-231, MDA-MB-435 and MCF-7 cells were transduced with viral supernatant supplemented with 8 μg/mL of Polybrene (Sigma-Aldrich). After 24 h, cells were selected in medium containing 0.6 μg/mL of puromycin (Sigma-Aldrich).

### Luciferase reporter plasmid constructs and assays

To construct the *WWTR1*-HRE and *SIAH1*-HRE reporters, 55-bp double-stranded oligonucleotides were inserted between the *Bam*HI and *Sal*I sites of pGL2-Promoter (Promega). All plasmid constructs were confirmed by nucleotide sequence analysis. A 247-bp CTGF promoter sequence was amplified from human genomic DNA by PCR (primers: 5′-CCCCTCGAGAGTGTGCCAGCTTTTTCAGAC-3′ and 5′-CGAAGCTTCGAGCTGGAGGGTGGAGT-3′), purified by gel extraction, and inserted into the XhoI and HindIII sites of pGL2-Basic (Promega). For HRE reporter assays [48], cells were seeded onto 48-well plates and co-transfected with recombinant pGL2-Promoter plasmid, which contained HRE-WT or HRE-MUT sequences, and pSV-Renilla. Transfected cells were exposed to 20% or 1% O_2_ for 24 h. Firefly luciferase and Renilla luciferase activities in cell lysates were determined using the Dual-Luciferase Assay System (Promega).

### RT-qPCR

Total cellular RNA was extracted using TRIzol (Invitrogen), precipitated with isopropanol, treated with DNase I (Ambion), and reverse transcribed with the iScript cDNA Synthesis kit (Bio-Rad). qPCR analysis was performed using SYBR Green and the iCycler Real-time PCR Detection System (BioRad). The 2^− ΔΔCt^ method was used to calculate the relative gene expression [21]. Primer sequences are listed in Table S1.

### Subcellular fractionation

MDA-MB-231 subclones were resuspended in hypotonic buffer [10 mM HEPES/KOH (pH 7.5), 1 mM K_2_EDTA, 10 mM KCl, 1.5 mM MgCl_2_, 1 mM EGTA, 0.1% Igepal, 1 mM DTT, and protease inhibitor cocktail (Roche)], incubated on ice for 30 min, and disrupted in a Dounce homogenizer (60 strokes). The nuclei were collected by centrifugation at 800 *g* for 10 min, washed twice with isotonic buffer (hypotonic buffer plus 250 mM sucrose) and lysed in isotonic buffer by sonication. The supernatant was reserved as the cytosolic fraction.

### Immunoprecipitation and immunoblot assays

Whole cell lysates (WCLs) were prepared in RIPA lysis buffer. For co-immunoprecipitation assays, 30 μl of protein G-Sepharose beads (GE Healthcare) and immunoprecipitating antibody were incubated with 0.75 mg of WCL overnight at 4°C. Beads were washed 5 times in lysis buffer. Proteins were eluted in SDS sample buffer and separated by SDS-PAGE. Antibodies used in immunoblot and co-immunoprecipitation assays were: HIF-1α (BD Transduction Laboratory); HIF-2α, TAZ, LATS2, LATS1, SIAH1, histone H3, α-tubulin, IgG (Novus Biologicals); and β-actin (Santa Cruz). HRP-conjugated anti-rabbit (Amersham) and anti-mouse (Santa Cruz) secondary antibodies were used. Chemiluminescent signal was developed using ECL Plus (GE Healthcare). For ubiquitination assays, cells were co-transfected with vector encoding His-tagged ubiquitin, and shSI1 or NTC vector, and exposed to 1% O_2_ for 48 h with 10 μM MG132 added for the last 6 h. Total ubiquitinated proteins were precipitated from WCLs by Ni-NTA beads (Qiagen) and subjected to immunoblot assays.

### ChIP assay

ChIP assays were performed as previously described [18]. HCC1954 and MCF-7 cells were cross-linked in 3.7% formaldehyde for 10 min and lysed with SDS lysis buffer. Chromatin was sheared by sonication and lysates were pre-cleared with salmon sperm DNA/protein A-agarose slurry (Millipore) and incubated with IgG (Novus Biologicals) or antibodies against the following proteins: HIF-1α (Santa Cruz), HIF-2α, HIF-1β and TAZ (Novus Biologicals). Salmon sperm DNA/protein A-agarose slurry was added and the agarose beads were washed sequentially with: low- and high-salt immune complex wash buffers; LiCl immune complex wash buffer; and twice with TE buffer (10 mM Tris-HCl/1 mM EDTA). DNA was eluted in 1% SDS with 0.1 M NaHCO_3_, and crosslinks were reversed by addition of 0.2 M NaCl. DNA was purified by phenol-chloroform extraction and ethanol precipitation, suspended in 50 μl TE buffer, and 2-μl aliquots were used for qPCR. Primer sequences are listed in Table S1.

### Immunohistochemistry

Tumors were fixed in 10% formalin and paraffin embedded. Sections were dewaxed in xylene, hydrated with graded ethanol, followed by antigen retrieval using citrate buffer (pH 6.1). The LSAB+ System HRP kit (DAKO) was used with antibodies against TAZ and P4HA1 (Novus Biologicals). Sections were counterstained with Mayer's hematoxylin (Sigma). HIF-1α immunohistochemistry was performed as described [49].

### Immunofluorescence

MDA-MB-231 cells plated on Lab-Tek II chamber slides (Thermo Fisher Scientific) were fixed with 4% paraformaldehyde (Sigma), permeabilized with 0.1% Triton X-100 (Fisher) for 5 min, blocked with PBS supplemented with 10% BSA for 20 min, and stained with anti-TAZ antibody (Novus Biologicals) overnight at 4°C, followed by incubation with FITC-conjugated goat anti-rabbit IgG (Novus Biologicals). F-actin and nuclear DNA were stained using Alexa Fluor 568-conjugated phalloidin (Life Technologies) and 300 nM DAPI (Invitrogen), respectively. Fluorescent imaging was performed using a 63× (1.4 NA), oil immersion objective on an AxioObserver microscope (Carl Zeiss), which was equipped with ApoTome.2 and an ORCA-ERG digital camera (Hamamatsu). Images were processed with ZEN software 2012 (Carl Zeiss). Fluorescence intensities of TAZ in nucleus and cytoplasm were determined using ImageJ software (NIH). Fifty cells were randomly imaged and scored for each experiment, and data from three independent experiments were plotted as mean + SEM.

### Aldefluor assay and flow cytometry

Cells were exposed to 20% or 1% O_2_ for 72 h and subject to Aldefluor assay (Stem Cell Technologies). Cells were suspended in assay buffer containing 1 μmol/L BODIPY-aminoacetaldehyde and incubated for 40 min at 37°C. As a negative control, for each sample an aliquot of cells was treated with the ALDH inhibitor diethylaminobenzaldehyde (50 mM). Samples were subjected to flow cytometry analysis (FACScalibur, BD Biosciences). The top and bottom 5% of Aldefluor expressing cells were sorted by FACS and considered ALDH^+^ and ALDH^−^, respectively, for RNA analysis.

### Mammosphere assays

Cells were exposed to 20% or 1% O_2_ in monolayer adherent culture for 72 h. Single cells were counted and plated on 6-well ultra-low attachment culture plates (Corning) at a density of 5000 cells/mL for primary mammosphere formation and 2000 cells/mL for secondary mammosphere formation in complete Mammocult medium (Stem Cell Technologies). Mammospheres (diameter ≥ 70 μm) were counted after 7 d. Mammosphere cultures were imaged using an Olympus phase-contrast microscope and mammosphere diameters were determined using ImageJ software. Mammospheres were collected by centrifugation at 800 rpm and enzymatically dissociated by incubation in trypsin-EDTA solution (Invitrogen) for 2 min at 37°C. Single cell suspensions were then counted and reseeded for secondary mammosphere formation.

### Orthotopic transplantation

Studies using 6- to 8-week-old female SCID mice (NCI) were performed according to protocols approved by the Johns Hopkins University Animal Care and Use Committee in accordance with the NIH Guide for the Care and Use of Laboratory Animals. 1×10^3^ cells of MDA-MB-231 subclones were suspended in 100 μl Matrigel (BD Biosciences) and injected into the mammary fat pad. Tumor formation was assayed by palpation.

### Survival analysis

Seven Affymetrix HG-U133A microarray datasets, which together totaled 1,098 breast cancer patient samples annotated with histological tumor grade and survival outcome, were downloaded from the NCBI Gene Expression Omnibus (http://www.ncbi.nlm.nih.gov/geo/): GSE1456 [50]; GSE3494 [51]; GSE4922 [52]; GSE6532 [53-55]; GSE2990 [56]; GSE7390 [57]; and GSE11121 [58]. The TAZ/YAP pathway signature has been previously reported as genes with expression induced > 2-fold in both TAZ-overexpressing and YAP-overexpressing MCF10A cells [8]. The HIF signature consists of 10 genes with mRNA expression induced by hypoxia in MDA-MB-231 cells in a HIF-dependent manner: *P4HA1, P4HA2, PLOD1, PLOD2, VEGFA, LOX, L1CAM, SLC2A1, CXCR3, PDGFB*. The mean expression for all genes in the signature was determined and patients were stratified by low (< median) versus high (> median) signature expression.

### Statistical analysis

Data are presented as mean ± SEM and were analyzed using an unpaired two-tailed Student's t-test for two groups or ANOVA followed by Bonferroni post-test for multiple groups. The Chi-square test was used for analyzing tumor formation. Survival data were analyzed using Kaplan Meier survival plots and *p* values were calculated using the Wilcoxon rank sum test. *P* values < 0.05 were considered significant.

## SUPPLEMENTARY MATERIAL, FIGURES AND TABLE


