# Supplementary material for: Hypoxia-inducible factor 1 mediates TAZ expression and nuclear localization to induce the breast cancer stem cell phenotype
Source: Oncotarget. 2014 Dec 18;5(24):12509–27. doi: 10.18632/oncotarget.2997 (PMC4350363; doi:10.18632/oncotarget.2997)
Supplement: Supplementary file 1 [file oncotarget-05-12509-s001.pdf]

Hypoxia-inducible factor 1 mediates TAZ expression and nuclear localization to induce the breast cancer stem cell phenotype

Supplementary Material

Table S1: Sequences of oligonucleotide primers used for RT-qPCR and ChIP assays.

Primers used in qRT-PCR assays	Sequence
Hs-TAZ-FWD	GATCCTGCCGGAGTCTTTCTT
Hs-TAZ-REV	CACGTCGTAGGACTGCTGG
Hs-CTGF-FWD	AAAAGTGCATCCGTACTCCCA
Hs-CTGF-REV	CCGTCGGTACATACTCCACAG
Hs-PAI-1-FWD	ACCGCAACGTGGTTTTCTCA
Hs-PAI-1-REV	TTGAATCCCATAGCTGCTTGAAT
Hs-Survivin-FWD	AGGACCACCGCATCTCTACAT
Hs-Survivin-REV	AAGTCTGGCTCGTTCTCAGTG
Hs-SIAH2-FWD	CCCTTCCTGCCTGCCTTCC
Hs-SIAH2-REV	TCAGTGTCTATTAGCCAGCCATCC
Hs-LATS2-FWD	ACCCCAAAGTTCGGACCTTAT
Hs-LATS2-REV	CATTTGCCGGTTCACTTCTGC
Hs-SIAH1-FWD	CACCAGCAGTTCTTCGCAATCG
Hs-SIAH1-REV	CAATCGTCGCCTATGACCATTTAGC
Hs-18S-FWD	GAGGATGAGGTGGAACGTGT
Hs-18S-REV	AGAAGTGACGCAGCCCTCTA

Primers used in ChIP assays	Sequence
SIAH1-HRE-FWD	TCATTGTGCAGTGGGAAGAA
SIAH1-HRE-REV	AACCCTAGGAAATGCCAGGT
CTGF-FWD	GGAGTGGTGCGAAGAGGATA
CTGF-REV	GCCAATGAGCTGAATGGAGT
WWTR1-HRE-FWD	GGTGTCCATGACCTCTGTTT
WWTR1-HRE-REV	TACACCAGATTGCCTCCTCC

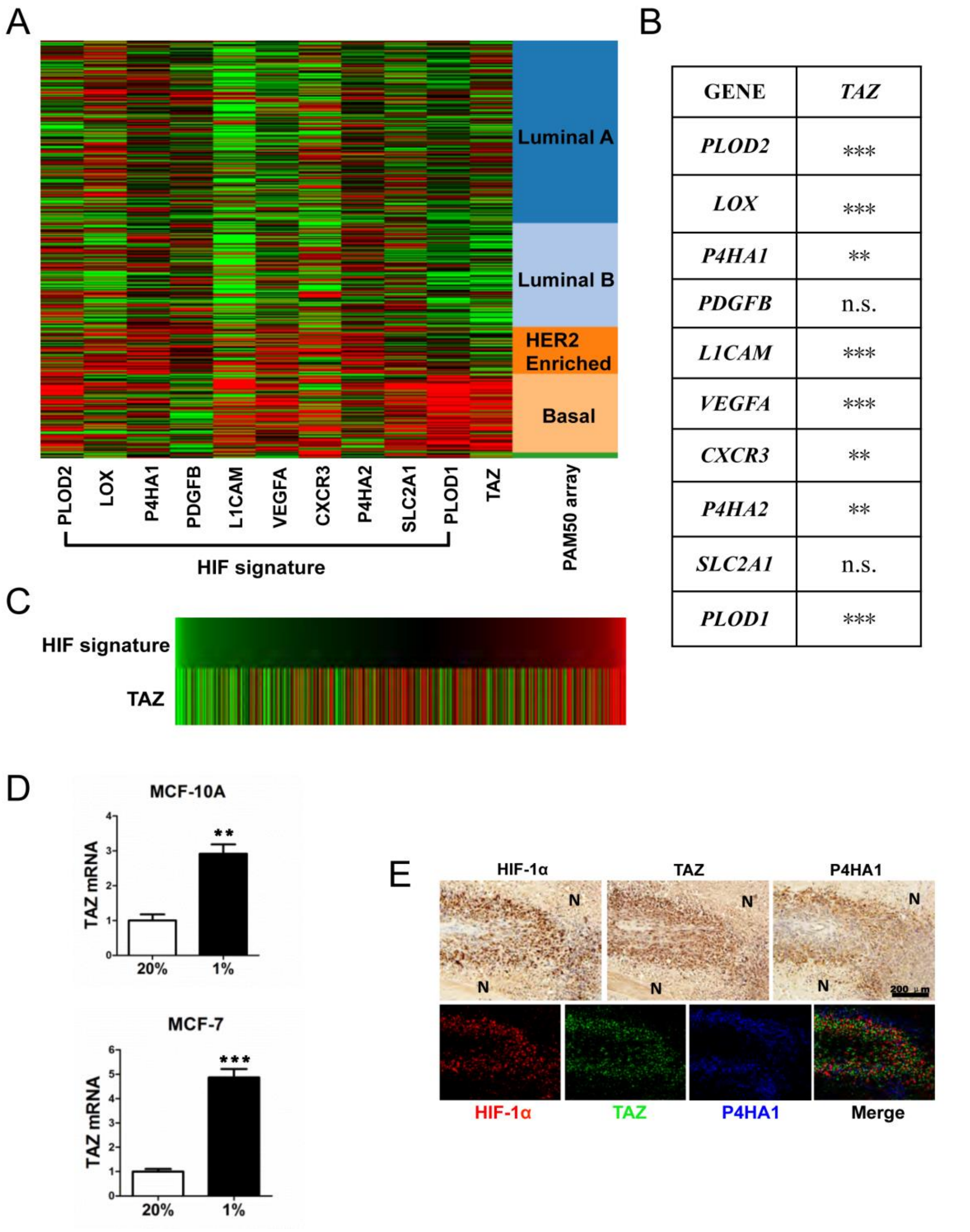

Figure S1: Analysis of TAZ mRNA expression. (A) The relative expression levels of 10 mRNAs encoded by HIF target genes and TAZ mRNA are shown for 1,160 breast cancer specimens from the Cancer Genome Atlas (TCGA), which were grouped according to the

expression of 50 mRNAs (PAM50; not shown) that define the breast cancer molecular subtypes (Luminal A, Luminal B, $HER2^+$, and Basal). (B) TAZ mRNA levels in each breast cancer specimen were compared with the expression HIF-regulated mRNAs using Pearson's correlation test. $^{**}P < 0.01$, $^{***}P < 0.001$; n.s., no significant positive correlation. (C) A HIF metagene signature, consisting of the combined expression of 10 HIF-regulated mRNAs in each breast cancer specimen, was generated and used to order the breast cancers from highest (red) to lowest (green) expression for comparison with TAZ mRNA expression. (D) Reverse transcription and quantitative real-time RT-PCR (RT-qPCR) was performed to quantify TAZ mRNA levels in breast cell lines following exposure to 20% or 1% O_2 for 24 h. For each sample, the expression of TAZ mRNA was quantified relative to 18S rRNA and then normalized to the result obtained from MCF-10A cells at 20% O_2 (mean ± SEM; n = 3). $^{**}P < 0.01$, $^{***}P < 0.001$ versus 20% O_2 (Student's *t* test). (This panel is an enlargement of the MCF-10A and MCF-7 data from the bar graph in Fig. 1A.) (E) Immunohistochemistry and light microscopy (top row) were performed to detect TAZ, P4HA1 and HIF-1α protein in MDA-MB-231 serial tumor sections. N, necrotic region. Image analysis was performed in which signals were pseudo-colored and then merged to show co-localization (bottom row).

A

GENE	*SIAH1*	*SIAH2*
PLOD2	***	*n.s.*
LOX	***	*n.s.*
P4HA1	*	*n.s.*
PDGFB	***	*n.s.*
L1CAM	***	*n.s.*
VEGFA	*n.s.*	*n.s.*
CXCR3	*n.s.*	*n.s.*
P4HA2	*n.s.*	*n.s.*
SLC2A1	**	*n.s.*
PLOD1	*n.s.*	*n.s.*

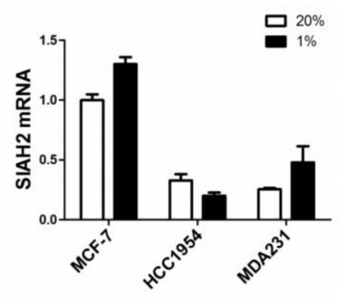

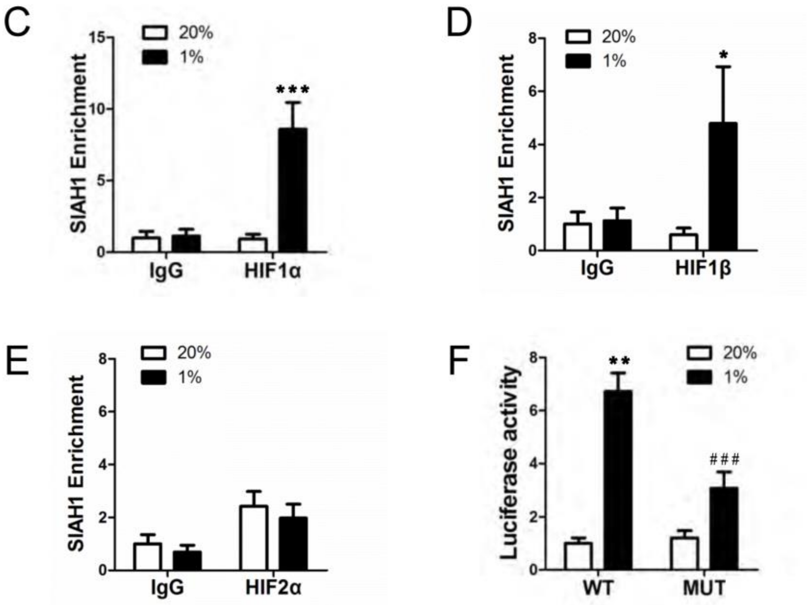

Figure S2: Regulation of *SIAH1* gene expression. (A) The expression of SIAH1 and SIAH2 mRNA in 1,160 breast cancer specimens from the TCGA database was compared with the expression of 10 HIF-regulated mRNAs using Pearson's correlation test. The asterisk(s) indicate significant positive correlations, as follows: $^{*}P < 0.05$, $^{**}P < 0.01$, $^{***}P < 0.001$; n.s., no significant positive correlation. (B) RT-qPCR was performed to

determine SIAH2 mRNA levels in breast cell lines following exposure to 20% or 1% O_2 for 24 h. For each sample, the expression of SIAH1 and SIAH2 mRNA was quantified relative to 18S rRNA and then normalized to the result obtained from MCF-7 cells at 20% O_2 (mean ± SEM; n = 3). (C-E) HCC1954 cells were exposed to 20% or 1% O_2 for 16 h and chromatin immunoprecipitation assays were performed using IgG or antibodies against HIF-1α (C), HIF-2α (D), or HIF-1β (E). Primers flanking the hypoxia response element (HRE) were used for qPCR and results were normalized to lane 1 (mean ± SEM; n = 3). $^{*}P < 0.05$, $^{***}P < 0.001$ versus 20% O_2 (Student's *t* test). (F) The *SIAH1* HRE containing a wild type (WT: 5'-G<u>CGT</u>GAACGG<u>CGT</u>G-3') or mutant (MUT; 5'-G<u>AAA</u>GAACGG<u>AAA</u>G-3') HIF-1 binding site was inserted into pGL2-Promoter (encoding firefly luciferase) and co-transfected with pSV-Renilla (encoding Renilla luciferase) into HCC1954 cells, which were incubated at 20% or 1% O_2 for 24 h. The firefly:Renilla luciferase ratio (Luciferase activity) was normalized to lane 1 (mean ± SEM; n = 3). $^{**}P < 0.01$ versus WT at 20% O_2; $^{###}P < 0.001$ versus WT at 1% O_2 (Student's *t* test).

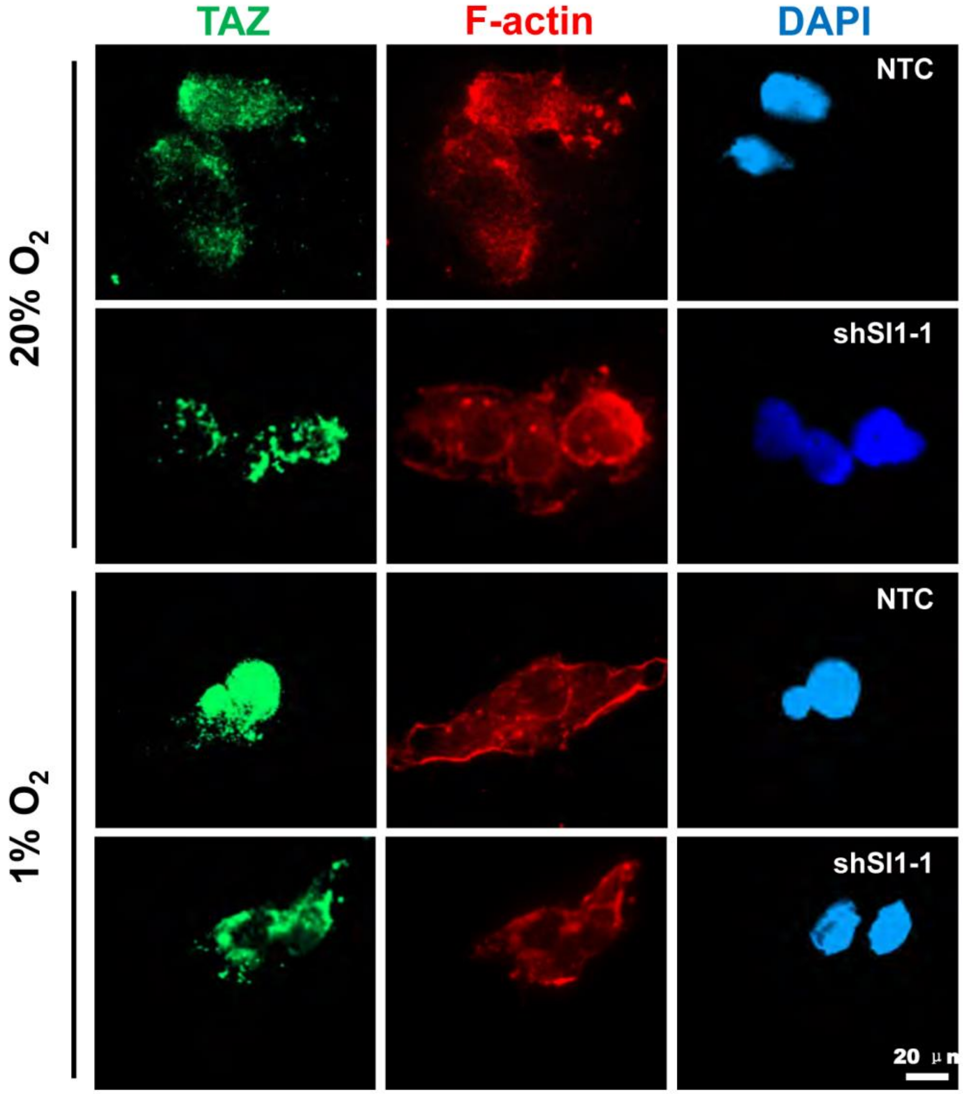

Figure S3: SIAH1 expression is required for hypoxia-induced nuclear localization of TAZ. MDA-MB-231 subclones (NTC and shSI1-1) were exposed to 20% or 1% O_2 for 48 h and stained with anti-TAZ antibody (green), Alexa Fluor 568-conjugated phalloidin to detect cytosolic F-actin (red), and DAPI to detect nuclear DNA (blue).
